# Clinical characteristics, organ failure, inflammatory markers and prediction of mortality in patients with community acquired bloodstream infection

**DOI:** 10.1186/s12879-018-3448-3

**Published:** 2018-10-26

**Authors:** Vu Quoc Dat, Nguyen Thanh Long, Vu Ngoc Hieu, Nguyen Dinh Hong Phuc, Nguyen Van Kinh, Nguyen Vu Trung, H. Rogier van Doorn, Ana Bonell, Behzad Nadjm

**Affiliations:** 1 Department of Infectious Diseases, Hanoi Medical University, no 1 Ton That Tung street, Dong Da district, Hanoi, Vietnam; 2 Wellcome Trust Major Overseas Programme, Oxford University Clinical Research Unit, Hanoi, 78 Giai Phong street, Dong Da district, Hanoi, Vietnam; 3 National Hospital for Tropical Diseases, 78 Giai Phong street, Dong Da district, Hanoi, Vietnam; 4 Department of Microbiology, Hanoi Medical University, no 1 Ton That Tung street, Dong Da district, Hanoi, Vietnam; 5 Nuffield Department of Clinical Medicine, Centre for Tropical Medicine, University of Oxford, Oxford, UK

**Keywords:** Bloodstream infection, Organ failure, Sequential organ failure assessment score, SOFA, qSOFA, Inflammatory markers, Procalcitonin, C-reactive protein

## Abstract

**Background:**

Community acquired bloodstream infection (CABSI) in low- and middle income countries is associated with a high mortality. This study describes the clinical manifestations, laboratory findings and correlation of SOFA and qSOFA with mortality in patients with CABSI in northern Vietnam.

**Methods:**

This was a retrospective study of 393 patients with at least one positive blood culture with not more than one bacterium taken within 48 h of hospitalisation. Clinical characteristic and laboratory results from the first 24 h in hospital were collected. SOFA and qSOFA scores were calculated and their validity in this setting was evaluated.

**Results:**

Among 393 patients with bacterial CABSI, approximately 80% (307/393) of patients had dysfunction of one or more organ on admission to the study hospital with the most common being that of coagulation (57.1% or 226/393). SOFA performed well in prediction of mortality in those patients initially admitted to the critical care unit (AUC 0.858, 95%CI 0.793–0.922) but poor in those admitted to medical wards (AUC 0.667, 95%CI 0.577–0.758). In contrast qSOFA had poor predictive validity in both settings (AUC 0.692, 95%CI 0.605–0.780 and AUC 0.527, 95%CI 0.424–0.630, respectively). The overall case fatality rate was 28%. HIV infection (HR = 3.145, *p* = 0.001), neutropenia (HR = 2.442, *p* = 0.002), SOFA score 1-point increment (HR = 1.19, *p* < 0.001) and infection with Enterobacteriaceae (HR = 1.722, *p* = 0.037) were independent risk factors for in-hospital mortality.

**Conclusions:**

Organ dysfunction was common among Vietnamese patients with CABSI and associated with high case fatality. SOFA and qSOFA both need to be further validated in this setting.

**Electronic supplementary material:**

The online version of this article (10.1186/s12879-018-3448-3) contains supplementary material, which is available to authorized users.

## Background

Bloodstream infection (BSI) is a common cause of sepsis and is associated with significant morbidity and in-hospital mortality worldwide [1]. It is ranked the 11th leading cause of death among adults in USA in 2014, with an age-adjusted death rate of 10.7 per 100,000 standard population [2]. In South and Southeast Asia, the incidence rate of community-acquired BSI in the period of 2004–2010 increased from 16.7 to 38.1 per 100,000 people per year and the 30 days mortality rate can reach up to 37.5% [3].

In patients with BSI, an increasing number of organs with dysfunction is correlated with increased morbidity and mortality [4]. Multiple organ dysfunction is a leading cause of morbidity and mortality in patients admitted to intensive care units (ICUs) in Europe, with an in-hospital mortality of 34.2% [5]. Sequential organ failure assessment (SOFA) score, and the related qSOFA (quickSOFA) score have been recently recommended for identifying sepsis and predicting outcome by the Third International Consensus Definitions for Sepsis and Septic Shock (Sepsis-3) [6]. qSOFA was originally designed for use outside the ICU, but it’s simplicity, brevity and lack of laboratory results, make it compelling for use in emergency departments and resource-constrained setting. However the validation of qSOFA is not consistent among studies quantifying the risk of death in those presenting with suspected infection in critical care [6–9]. Development and validation of these scores were mostly carried out in high income countries, with limited data on their validity in low- and middle income countries (LMICs) [7, 8, 10]. Additionally there have been few studies looking specifically at patients with BSI, a population with an associated increase in mortality.

This study aims to describe the clinical manifestations and associated organ dysfunctions as described by Sequential [Sepsis-related] Organ Failure Assessment (SOFA) scores and its correlation with mortality in patients with community acquired bloodstream infection at the time of presenting to a large teaching hospital in Vietnam, and their associated mortality.

## Methods

### Study design

This was a retrospective, cohort study of patients hospitalised at the National Hospital for Tropical Diseases (NHTD) (a tertiary referral infectious disease hospital) in northern Vietnam between January 2011 and December 2013. As a referral centre, this hospital often receives patients with specific infections (eg. central nervous system infections), complicated infections and those with severe infections who have failed on treatment elsewhere. Additionally, at the time of the study, the hospital had not establish a separate emergency department and intensive care unit (ICU), therefore we refer to the critical care unit (CCU) for the unit with both ventilated and unventilated beds, available haemodynamic support and renal replacement therapy. A convenience sampling method was used to select medical notes from the list of all hospitalised patients with positive bacterial blood cultures during the study period. The inclusion criteria were having a blood culture, taken within 48 h of hospitalisation (to any institution) for the current admission, positive for a recognised pathogen according to the US CDC’s National Healthcare Safety Network (NHSN) list [11]. Patients with infection with more than one bacterium were excluded, as were cases considered to be pseudobacteraemia [12].

### Data collection

Data was extracted from patients’ medical notes using a case-report form that captured patient demographics, reported history of prior medical illness, clinical manifestations, laboratory results, inflammatory markers within the first 24 h of admission to the study hospital and outcome at hospital discharge.

BSI with concurrent meningitis was defined in bacteremic patients who had cerebrospinal fluid examination within 24 h of blood drawn for microorganism isolation showing at least one of the following criteria: (1) turbid appearance; (2) leukocytosis (> 100 cells/mm3) or (3) leukocytosis from 10 to 100 cells/ mm3) and either an elevated protein (> 100 mg/dl) or decreased glucose (< 40 mg/dl) [13]. BSI with concurrent pneumonia was confirmed by radiology within 24 h of blood drawn for microorganism isolation. Gastrointestinal tract infection and urine tract infection were defined by the CDC/NHSN Surveillance Definitions for Specific Types of Infections [14]. Sequential [Sepsis-related] Organ Failure Assessment (SOFA) score, and quick SOFA (qSOFA) score were calculated using the worst parameters recorded within the first 24 h of admission to the study hospital and missing values were considered to be normal [6]. Organ dysfunction was defined by organ-specific SOFA scores ≥1. Failure of kidney function was further evaluated using the RIFLE criteria with RIFLE-F (Failure) defined as patients with a serum creatinine greater than three times the age adjusted upper limit of serum creatinine [15]. Neutropenia was defined as an absolute neutrophil count < 1500 cells/mm^3^, severe anemia as hemoglobin concentration was < 80 g/L and thrombocytopenia as a platelet count below 100 × 10^3^ cells/mm^3^.

The outcome at hospital discharge was defined as death for those who died in hospital or were palliatively discharged (discharged home for palliative care with the expectation of an early death, as per common practice in Vietnam) and ‘survived’ in all other cases.

### Data analysis

Data was analysed using IBM SPSS Statistics for Windows (IBM Corp., Armonk, NY). Depending on the distribution, continuous data were presented as mean (95% confidence interval) or median (interquartile range) and categorical data as number (percentage). To evaluate the predictive value of SOFA, qSOFA score, white blood cell counts, C-Reactive Protein (CRP) and procalcitonin levels, a receiver operating characteristic (ROC) curve and the area under the curve (AUC) were calculated along with the sensitivity, specificity, positive and negative predictive values, positive and negative likelihood ratios associated with the cut-off value that gave the highest difference between sensitivity and (1-specificity) (Youden index). Since procalcitonin level was obtained by a semi-quantitative test that was only quantitatively measured for levels under 100 ng/mL, the result of “above 100 ng/mL” was considered as 100 ng/mL. The Mann Whitney U test and Kruskal Wallis test were used to analyze continuous variables and the Chi-square and Fisher’s exact were used for bivariate analyses as appropriated. Logistic regression models were used to calculate unadjusted odds ratios (ORs) and 95% confidence intervals (CIs) for associations between clinical, laboratory characteristics and case fatality rate. Comparison of AUC between different ROC curves was performed using a nonparametric approach [16]. Cox proportional hazards regression was used to identify variables that predicted clinical outcomes. Variables for inclusion were selected by review of the literature (age, HIV infection status, neutropenia, SOFA score and aetiology of CABSI). All tests were two-tailed and differences were considered statistically significant at *p* values ≤0.05.

## Results

Among 400 patients with community acquired bloodstream infections (CABSI) there were 393 patients with infection with one bacterium included in this analysis, 7 dual infection cases were excluded (including 3 HIV infected cases with co-infection of *T. marneffei* and *S. aureus*, *Escherichia hermannii* or *Salmonella group D*; 1 case with *S. aureus* and *K. pneumoniae*, 1 case with *Enterococcus faecalis* and viridans streptococci, 1 case with *E. coli* and *K. pneumoniae* and 1 case with *S. aureus* and *E. coli* co-infection). Gram-negative bacteria dominated (70.7%, 278/393), comprising *Enterobacteriaceae* (50.9%, 200/393) and non-*Enterobacteriaceae* Gram-negative bacteria (19.8%, 78/393) followed by Gram-positive bacteria 29.3% (115/393).

### Clinical characteristics, aetiology of CABSI and organ failure

The median age of patients included was 48 years (IQR 36–60), with 271 males and a male to female ratio of 2.2:1. There was a history of chronic disease in 27% of patients, with the highest prevalence in patients with *Enterobacteriaceae* BSI, 34% (38/200). Thirty-eight percent (150/393) of patients were transferred from another hospital (< 48 h) for the current illness episode. The median time from onset of illness to hospitalisation at the study site was 5 days and 36.9% (145/393) were admitted directly to critical care.

Concurrent meningitis was confirmed in 18.3% (72/393) and pneumonia in 24.9% (98/393) of patients, with both conditions occurring in 2.8% (11/393) of patients with CABSI. Gram-positive organisms were isolated from 72.2% (52/72) of those with meningitis, *Enterobacteriaceae* in 18.1% (13/72) and non-*Enterobacteriaceae* Gram-negative bacteria in 9.7% (7/72), see Table 1 for details. Concurrent meningitis was found in 6.5% (13/200) patients with *Enterobacteriaceae* BSI, 9.0% (7/71) patients with non-*Enterobacteriaceae* Gram-negative bacteria BSI and 45.2% (52/115) patients with gram positive BSI. In the 98 cases of pneumonia the causative pathogens isolated from blood were *Enterobacteriaceae* (46.9%, 46/98), non-*Enterobacteriaceae* Gram-negative bacteria (25.5%, 25/98) and Gram-positive bacteria (27.6%, 27/98) (see Additional file 1: Table S1).

**Table 1:** Clinical characteristic on admission of patients with bloodstream infection

Factor	Proportion	Case fatality rate	Unadjusted odds ratios (95%CI) for case fatality	*P* values
Age (yrs)				
≤ 40 years old	122/393 (31%)	27 (22.1%)	1	
41–55 years old	152/393 (38.7%)	46 (30.3%)	1.527 (0.881–2.646)	0.131
≥ 56 years old	119/393 (30.3%)	37 (31.1%)_	1.588 (0.891–2.828)	0.117
Male sex (%)	271/393 (69.0%)	84 (31.0%)	1.659 (1.002–2.746)	0.049
Any previous hospitalisation (%)	150/393 (38.2%)	62 (41.3%)	2.862 (1.819–4.503)	< 0.001
Any antibiotic prior to NHTD hospitalisation (%)	50/150 (33.3%)	23 (46.0%)	1.332 (0.671–2.646)	0.412
Time from onset to current hospitalisation < 5 days	217/393 (55.2%)	57 (26.3%)	0.827 (0.532–1.286)	0.399
Direct ICU admission	145/393 (36.9%)	68 (46.9%)	4.331 (2.720–6.898)	< 0.001
Any history of medical disease	106/393 (27.0%)	44 (41.5%)	2.376 (1.479–3.818)	< 0.001
HIV	19/393 (4.8%)	10 (52.6%)	3.044 (1.202–7.710)	0.019
Moderate or severe liver disease	53/393 (13.5%)	25 (47.2%)	2.679 (1.481–4.844)	0.001
Diabetes	25/393 (6.4%)	7 (28.0%)	1.001 (0.406–2.466)	0.999
Concurrent foci of infection				
Radiology-confirmed pneumonia on admission	98/393 (24.9%)^a^	29 (29.6%)	1.11 (0.671–1.837)	0.684
Lumbar puncture confirmed meningitis on admission	72/393 (18.3%)^b^	19 (26.4%)	0.906 (0.509–1.614)	0.738
Heart valve vegetations during hospitalisation	16/393 (4.1%)	4 (25.0%)	0.852 (0.269–2.701)	0.786
Any abscess during hospitalisation	33/393 (8.4%)^c^	6 (18.2%)	0.547 (0.219–1.364)	0.196
Organ dysfunction on admission				
Cardiovascular	65/393 (16.5%)	52 (80.0%)	18.621 (9.522–36.415)	< 0.001
Respiratory	87/393 (22.1%)	53 (60.9%)	6.81 (4.057–11.431)	< 0.001
CNS	105/393 (26.7%)	55 (52.4%)	4.66 (2.876–7.551)	< 0.001
Hepatic	146/393 (37.2%)	57 (39.0%)	2.344 (1.494–3.678)	< 0.001
Renal	153/393 (38.9%)	66 (43.1%)	3.379 (2.139–5.339)	< 0.001
Coagulation	226/393 (57.5%)	85 (37.6%)	3.424 (2.070–5.663)	< 0.001

^a^Isolates from blood in patients with pneumonia were *K. pneumoniae* (22.4%, 22/98), *E. coli* (16.3%, 16/98), *S. maltophilia* (11.2%, 11/98), *Burkholderia pseudomallei* (8.2%, 8/98), *S. aureus* (7.1%, 7/98) and *S. suis* (7.1%, 7/98) and other pathogens (23.5%, 27/98)

^b^Isolates from blood in patients with meningitis were *S. suis* (40/72, 55.6%), *K. pneumoniae* (8/72, 11.1%), *Stenotrophomonas maltophilia* (7/72, 9.7%), *S. aureus* (5/72, 6.9%). *Enterococcus* species (2/72, 2.8%), *Listeria* species (2/72, 2.8%), *E. coli* (2/72, 2,8%), *Salmonella enterica (2/72,2.8%)* and each of *S. pneumoniae,* beta hemolytic *Streptococcus, viridans* group *Streptococcus* and *Enterobacter* species (1/72, 1.4%)

^c^There were 16 cases with liver abscess with isolates from blood were *K. pneumoniae* (8/16 or 50%), *E. coli* and *Salmonella enterica* (2/16 of each, or 12.5%), *Aeromonas* species, *Enterobacter* species, *S. suis* and *viridans* group *streptococci* (1/16 of each, 6.3%)

A further 8.4% (33/393) of patients presented with or developed at least one abscess, of which 2 patients (6.1%) had 2 abscess foci and 1 patient (3.0%) had 3 abscess foci in different locations. The locations were 16/37 (43.2%) liver, 8 (21%) skin, 4 (10.8%) muscle, 3 (8.1%) brain, 3 (8.1%) spleen, 2 (5.4%) lung, 1 (2.7%) eyelid. Endocarditis was confirmed by echocardiography in 4.1% (16/393) patients. These were due to *Staphylococcus aureus* (7/16, 43.8%), viridans group *streptococci* (4/16, 28.6%), *Enterococcus* species (3/16, 18.8%), *Klebsiella pneumoniae* (1/16, 6.25%) and *Pseudomonas aeruginosa (*1/16, 6.25%). The distribution of aetiology by the foci of infection is presented in Additional file 1: Table S1*.*

On the day of admission to the study hospital, BSI patients had a median SOFA score of 3 (IQR 1–7) and 78.1% (307/393) of patients had dysfunction of at least one organ. The SOFA score differed significantly between patients admitted direct to CCU (median of 7, IQR 4–12) and medical wards (median of 2, IQR 0–4) (*p* < 0.001). *Enterobacteriaceae* BSIs accounted for most cases with SOFA score above 12 (74% or 28/38, vs. 48% (172/355) in patients with SOFA score ≤ 12, *p* = 0.003) or more than 3 organ dysfunctions (60% or 47/78, vs. 49% or 153/315 in patients with ≤3 organ dysfunctions, *p* = 0.065). qSOFA score was ≥2 in 28.6% (71/248) of patients that were initially admitted to medical wards. The unadjusted associations between clinical factors and case fatality are presented in Table 1.

### Laboratory results and inflammatory markers

The proportions of patients with white blood cells count < 4 × 10^9^/l, 4–12 × 10^9^/l and > 12 × 10^9^/l were 11.8% (46/389), 47% (183/389) and 41.1% (160/389) respectively. Neutropenia, severe anaemia and thrombocytopenia was presented in 7.2% (28/388), 8% (31/389) and 43.4% (169/389) patients with BSI on admission to the study hospital, respectively. The proportion of patients with RIFLE-F, increased lactate, procalcitonin or CRP were not significantly different when classified by bacterial aetiology (*Enterobacteriaceae*, non-*Enterobacteriaceae* and Gram-positive). Lactate levels were only available for 73 patients and 52/73 (69.9%) patients had lactate level ≥ 2 mmol/L on admission. The median lactate level increased significantly with increasing SOFA score; from 1.23 mmol/L (IQR: 0.99–2.04 mmol/L) in those with a SOFA score < 6, to 7.89 mmol/L (IQR: 3.7–10.38) in those with a SOFA score > 12 (*p* < 0.001, Kruskal Wallis test). There was no significant difference between median lactate levels in those with qSOFA < 2 and qSOFA≥2 (*p* = 0.055, Mann-Whitney U test). Laboratory factors associated with case mortality was showed in Table 2. There was no unadjusted associated between procalcitonin and CRP levels with case fatality rates.

**Table 2:** Laboratory characteristics on admission

	Proportion	Case fatality	Unadjusted odds ratios (95%CI) for case fatality	*P* values
Neutropenia (< 1500 cell/mm^3^) (%)	28/388 (7.2%)	22 (78.6%)	11.682 (4.588–29.746)	< 0.001
Hemoglobin< 80 g/L	31/389 (8.0%)	11 (35.5%)	1.459 (0.675–3.156)	0.337
RIFLE criteria				
No renal dysfunction	283/386 (73.3%)	56 (19.8%)	1	
RIFLE-risk	50/386 (13.0%)	16 (32.0%)	1.908 (0.984–3.699)	0.056
RIFLE-failure	52/386 (13.5%)	35 (67.3%)	8.346 (4.361–15.971)	< 0.001
Hypoalbuminemia (albumin ≤30 g/L)	72/245 (29.4%)	39 (54.2%)	3.263 (1.839–5.789)	< 0.001
Aspartate Aminotransferase (AST) ≥ 2 ULN	153/379 (40.4%)	65 (42.5%)	3.435 (2.150–5.487)	< 0.001
Alanine aminotransferase (ALT) ≥ 2 ULN	109/378 (28.8%)	45 (41.3%)	2.449 (1.520–3.947)	< 0.001
Platelet < 100 × 10^3^/mm^3^	169/389 (43.4%)	70 (41.4%)	3.282 (2.068–5.208)	< 0.001
Procalcitonin				
PCT ≤ 0.005 ng/mL (%)	6/239 (2.5%)	2 (33.3%)	1	
PCT > 0.005–2 ng/mL (%)	83/239 (34.7%)	11 (13.3%)	0.306 (0.050–1.871)	0.2
PCT > 2–10 ng/mL (%)	59/239 (24.7%)	20 (33.9%)	1.026 (0.173–6.087)	0.978
PCT > 10–100 ng/mL (%)	69/239 (28.9%)	29 (42.0%)	1.45 (0.249–8.457)	0.68
PCT > 100 ng/mL (%)	22/239 (9.2%)	14 (63.6%)	3.5 (0.520–23.559)	0.198
C-reactive protein (CRP) (median, IQR) (mg/L)				
CRP less than 5 mg/L (%)	14/341 (4.1%)	4 (28.6%)	1	
CRP from 5.01 to 20 mg/L (%)	26/341 (7.6%)	4 (15.4%)	0.455 (0.094–2.195)	0.326
CRP from 20.001 to 100 mg/L (%)	112/341 (32.8%)	29 (25.9%)	0.873 (0.254–3.001)	0.83
CRP more than 100 mg/L (%)	189/341 (55.4%)	56 (29.6%)	1.053 (0.317–3.498)	0.933

*ULN* upper limit of normal; *RIFLE* Risk, Injury, Failure, Loss of kidney function, and End-stage kidney disease

### Mortality and associated factors

The overall case-fatality of CABSI was 28% (110/393), of which 71.8% (79/110) occurred within 7 days of admission to the study hospital. Case fatality rates in patients with CABSI due to *Enterobacteriaceae*, non-*Enterobacteriaceae* Gram-negative bacteria and Gram-positive bacteria were 33.5%, 25.6% and 20%, respectively. Among the most common isolates, the case fatality was 35.2% (31/88) in *K. pneumoniae*, 32.8% (21/64) in *Escherichia coli*, 9.3% (4/43) in *Streptococcus suis*, 15.4% (6/39) in *Stenotrophomonas maltophilia* and 32.4% (11/34) in *S. aureus*. The case-fatality in patients directly admitted to CCU was 46.9% (68/145).

**Fig. 1:**
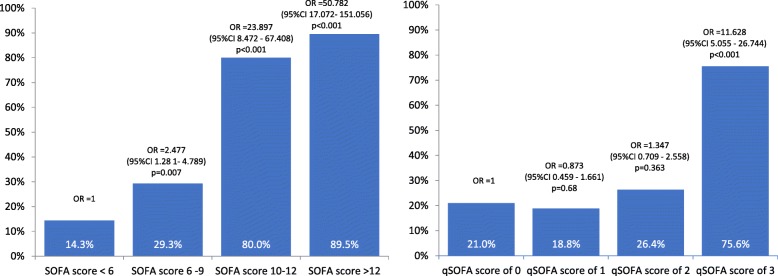
Case fatality rate by the SOFA and qSOFA scores within the first 24 h of admission

Organ dysfunction was associated with higher risk of in-hospital mortality (33.2% patients with at least one organ dysfunction on admission to the study hospital vs 9.3% patients without, *p* < 0.001). Case fatality rate increased with increasing SOFA score (Fig. 1). The mortality in patients with 1, 2, 3 and more than 4 organs dysfunction was 17% (17/100), 19.5% (16/82), 31.9% (15/47) and 69.2% (54/78), respectively. The highest case fatality rates were observed in patients with cardiovascular, respiratory and central nervous system (CNS) dysfunction, 80% (52/65), 60.9% (53/87), 52.4% (55/105), respectively. qSOFA < 2 was associated with lower mortality compared with qSOFA ≥2, 18.8% (26/138) vs 40.6% (63/155), *p* < 0.001. Among patients admitted directly to CCU, SOFA performed well at predicting in-hospital mortality (AUC 0.858, 95%CI 0.793–0.922) while qSOFA was a poor predictor (AUC 0.692, 95%CI 0.605–0.780) in this population. However, outside of CCU, regardless of eventual CCU admission, both SOFA and qSOFA had poor predictive validity (AUC 0.667, 95%CI 0.577–0.758 and AUC 0.527, 95%CI 0.424–0.630, respectively).

Table 3 shows the prognostic validity of the Youden index of SOFA, qSOFA, WBC, CRP and PCT on admission to NHTD in all patients. The SOFA score was more accurate than qSOFA in predicting mortality (AUC = 0.795 vs 0.658, *p* < 0.001); PCT (AUC = 0.703), and WBC (AUC = 0.642) was more accurate than CRP (AUC = 0.561) in predicting mortality (*P* < 0.001 and *P* = 0.0498 respectively).

**Table 3:** Performance of initial SOFA score, qSOFA score, white blood cells, C-reactive protein and procalcitonin, in prediction of in-hospital mortality

	SOFA (*n* = 393)	qSOFA (*n* = 393)	WBC (*n* = 389)	CRP (*n* = 341)	Procalcitonin (*n* = 239)
AUC (95% CI)	0.795 (0.741–0.850)	0.658 (0.595–0.721)	0.642 (0.576–0.708)	0.561 (0.492–0.630)	0.703 (0.629–0.776)
Cut-off value	≥ 9	≥ 3	≤ 7.39	≥ 210	≥ 5.49
Sensitivity	53.6%	30.9%	77.9%	35.5%	69.7%
Specificity	94.7%	96.1%	49.5%	75.8%	63.2%
Positive predictive value	79.7%	75.6%	79.9%	35.5%	46.9%
Negative predictive value	84.0%	78.2%	46.6%	75.8%	81.8%
Positive likelihood ratio	10.1194	7.9521	1.5430	1.4667	1.8945
Negative likelihood ratio	0.4896	0.7189	0.4470	0.8511	0.4789

*SOFA* Sequential Organ Failure Assessment, *qSOFA* quick Sequential Organ Failure Assessment, *WBC* white blood cell, *CRP* C-reactive protein

In unadjusted association analysis, higher mortality was associated with male sex, any previous hospitalisation, direct CCU admission, history of HIV infection, moderate severe liver diseases and. Analysis, male sex, previous hospitalisation prior to NHTD admission, any history of chronic disease, history of moderate or severe liver diseases, HIV infection, organ dysfunctions on admission, neutropenia, haemoglobin < 80 g/L, RIFLE failure (RIFLE-F), hypoalbuminemia (< 30 g/L), elevated liver enzymes (> 2 times the upper limit of normal), thrombocytopenia (< 100 × 10^3^/mm^3^) and were associated with increased in-hospital mortality (*p* values < 0.05). In Cox regression proportional hazards model, HIV infection (HR = 3.145, *p* = 0.001), neutropenia (HR = 2.442, *p* = 0.002), SOFA score 1-point increment (HR = 1.19, *p* < 0.001) and infection with *Enterobacteriaceae* (HR = 1.722, *p* = 0.037) were significant risk factors for in-hospital mortality (Table 4).

**Table 4:** Cox proportional hazards model of factors associated with all-cause in-hospital case fatality

Variable	Hazard ratio (95% CI)	*P*-value
Age (each increase of 1 year)	1.006 (0.994–1.019)	0.321
HIV infection	3.145 (1.569–6.305)	0.001
Absolute neutrophil count < 1500 cells/mm^3^	2.442 (1.381–4.319)	0.002
SOFA score (each increase of 1 point)	1.190 (1.146–1.235)	< 0.001
Aetiology of CABSI (gram-positive bacterial infection as reference)		
*Enterobacteriaceae* infections	1.722 (1.034–2.869)	0.037
Non *Enterobacteriaceae* Gram-negative infections	1.528 (0.824–2.834)	0.178

## Discussion

This retrospective study describes the clinical characteristics and outcomes in this high-risk group of patients with community acquired BSI. Multi-organ dysfunction and case-fatality rates were high in all aetiological bacterial groups. SOFA score on CCU admission had good prognostic accuracy for in-hospital mortality whilst qSOFA, WBC, CRP and PCT did not.

BSI patients admitted directly to CCU in our study had a median SOFA score of 7 (IQR 4–12), comparable with large-scale validation studies of Sepsis-3 criteria in the US (median of 6, IQR 3–9) [10] and Australia and New Zealand (median of 5, IQR 3–8) [7]. The proportion of bacteraemic patients with qSOFA of 2 or above in our study (39.4%) was also higher than in studies of sepsis conducted in high income countries (10–27%) [8, 10]. The percentage of patients with pneumonia in our CABSI cohort (24.9%) was lower than or similar to other studies on CABSI (24–38%) [17–19]. *K. pneumoniae* was the most common pathogen isolated from bacteremic patients with pneumonia in our study, reflecting its role here as an important cause of community acquired pneumonia [20]. We also confirmed the role of *S. suis* as the leading pathogen causing BSI associated with meningitis in Viet Nam [21]. Our findings further confirmed the reduction of *Neisseria meningitidis* in Viet Nam which was reported in 0.5% of blood isolates [22] and around 4.4% of cerebral spinal fluid (CSF) isolates before 2005 [23]. We also report the high prevalence of *Enterobacteriacaea* (18.1% or 13/72) as a cause of meningitis in adult BSI patients compared to previous studies from Viet Nam (13.5% or 30/222 of CSF isolates from 1996 and 2005) [23], Iceland (11.3% or 12/106 of positive CSF cultures during 1995–2010) [24] and Denmark (6.1% or 88/1437 during 1991–2000) [25]. The high prevalence of meningitis associated with *Enterobacteriaceae* in this setting may be related to *Strongyloides* hyperinfection, given the evidence for high seroprevalence of *Strongyloides* infection in this population [26].

A review of CABSI in south and Southeast Asia from 1990 to 2010 showed the most frequent isolates in adult patients were *Salmonella enterica* (37.8%), *S. aureus* (12.6%) and *E. coli* (12%) with an overall case fatality rate of 9% [27]. In North America and Europe, there was a significant increase in bloodstream infection caused by Gram-negative bacteria, and case fatality rates in the period 1992–2008 were 13–20.6% in patients with CABSI [1]. The overall case fatality of 28% in our CABSI patients was lower than in a study in Thailand [3] from 2004 and 2010 (37.5%) where the most common pathogens were *E. coli* (23.1%), *Burkholderia pseudomallei* (19.3%), and *S. aureus* (8.2%) but higher than in Cambodia (22.1%) where there was a predominance of *E. coli* (29.7%), *Salmonella* spp. (14.4%) and *B. pseudomallei* (12.6%) in the period of 2007–2010) [28]. The higher case fatality in Thailand and our study may relate to the shift in the aetiology of CABSI from *Salmonella* to other Gram-negative bacteria observed since the last decade.

Organ dysfunction is strongly associated with in-hospital mortality. In a multicentre study of severe sepsis in Spain, case fatality in patients with more than 4 organs with dysfunction was 78.4% [29]. From a large prospective European study, case fatality in patients with more than 3 organs with dysfunction was 58% and the, highest in-hospital mortality rates were observed in patients with coagulation failure (45%) [5]. In high income settings, among ICU patients with suspected infection, the predictive accuracy for in-hospital mortality is higher using SOFA than qSOFA (AUC = 0.74; 95% CI, 0.73–0.76; vs AUC = 0.66; 95% CI, 0.64–0.68) whilst outside of ICU, the predictive validity of qSOFA (AUC = 0.81; 95% CI, 0.80–0.82) was better than SOFA (AUC = 0.79; 95% CI, 0.78–0.80; *P* < 0.001) [10]. In a prospective study of patients with suspected infection admitted to an emergency department in Norway, the qSOFA had poor performance to predict 7-day and 30-day mortality with AUCs < 0.6 in both multiple imputation and complete case analysis [30]. The usefulness of qSOFA in low- and middle income countries has not been well established. Procalcitonin levels can serve as a useful marker to rule out sepsis and discriminate contamination from true bloodstream infection [31, 32]. Our study shows a poor prediction of initial PCT and CRP in prediction of mortality.

Our study has some major limitations. Firstly, as the study site is a referral hospital specialising in infectious diseases, the aetiologies, clinical manifestations, severity and response to the treatment may be different from those presenting to a general hospital. Secondly, SOFA and qSOFA was calculated based on the worst parameters within 24 h of admission to the study hospital which may not accurately present the severity of infection at arrival. Thirdly, due to the retrospective design, the data collection was incomplete and unbalanced distribution of missing data can be a bias in the prediction models The utilisation of SOFA and qSOFA needs to be validated prospectively in other setting at different time points of assessment.

## Conclusions

In conclusion, community acquired BSI has a high rate of organ dysfunction and mortality in this setting. SOFA performed well at predicting those at risk of death admitted directly to CCU, whilst qSOFA performed poorly. Further prospective validation in low- and middle income settings is needed.

## Additional file

Additional file 1: **Table S1.** The aetiology of BSI by the foci of infection (DOCX 19 kb).

## Abbreviations

AUC: Area under the curve
BSI: Bloodstream infection
CABSI: Community acquired bloodstream infection
CCU: Critical care unit
CI: Confidence interval
CNS: Central nervous system
CRP: C-reactive protein
CSF: Cerebral spinal fluid
HR: Hazard ratio
ICU: Intensive care unit
IQR: Interquartile range
LMICs: Low- and middle income countries
NHSN: National Healthcare Safety Network
NHTD: National Hospital for Tropical Diseases
OR: Odds ratio
PCT: Procalcitonin
qSOFA: Quick sequential organ failure assessment
ROC: Receiver operating characteristic
SOFA: Sequential organ failure assessment
