# Supplementary material for: Clinical characteristics, organ failure, inflammatory markers and prediction of mortality in patients with community acquired bloodstream infection
Source: BMC Infect Dis. 2018 Oct 26;18:535. doi: 10.1186/s12879-018-3448-3 (PMC6204014; doi:10.1186/s12879-018-3448-3)
Supplement: Supplementary file 1 — Table S1. The aetiology of BSI by the foci of infection (DOCX 19 kb). [file 12879_2018_3448_MOESM1_ESM.docx]

Table S1: The aetiology of BSI by focus of infection

|  | Radiology-confirmed pneumonia on admission  N=98 | Lumbar puncture confirmed meningitis on admission  N=72 | Heart valve vegetations during hospitalization  N=16 | Any abscess during hospitalization  N=33 | Gastroenteritis or bile duct obstruction  N=182 | Urinary tract infections  N=19 |
| --- | --- | --- | --- | --- | --- | --- |
| Enterobacteriaceae | 46 (46.9%) | 13 (18.1%) | 1 (6.3%) | 18 (54.6%) | 100 (55.0%) | 15 (79.0%) |
| *Klebsiella pneumoniae* | 22 (22.4%) | 8 (11.1%) | 1 (6.3%) | 11 (33.3%) | 41 (22.5%) | 3 (15.8%) |
| *Escherichia coli* | 16 (16.3%) | 2 (2.8%) | 0 | 3 (9.1%) | 40 (22.0%) | 8 (42.1%) |
| *Salmonella typhy and paratyphi* | 0 | 0 | 0 | 0 | 2 (1.1%) | 0 |
| *Other Salmonella* | 3 (3.1%) | 2 (2.8%) | 0 | 2 (6.1%) | 7 (3.9%) | 2 (10.5%) |
| *Serratia marcescens* | 2 (2.0%) | 0 | 0 | 1 (3.0%) | 3 (1.6%) | 0 |
| *Enterobacter* species | 2 (2.0%) | 1 (1.4%) | 0 | 1 (3.0%) | 5 (2.7%) | 0 |
| Other *Enterobacteriaceae* species | 1 (1.0%) | 0 | 0 | 0 | 2 (1.1%) | 2 (10.5%) |
| Non-Enterobacteriacae | 25 (25.5%) | 7 (9.7%) | 1 (6.3%) | 8 (24.2%) | 27 (14.8%) | 2 (10.5%) |
| *Acinetobacter species* | 0 | 0 | 0 | 1 (3.0%) | 1 (0.5%) | 0 |
| *Aeromonas species* | 2 (2.0%) | 0 | 0 | 2 (6.1%) | 4 (2.2%) | 0 |
| *Burkholderia species* | 8 (8.2%) | 0 | 0 | 3 (9.1%) | 2 (1.1%) | 1 (5.3%) |
| *Stenotrophomonas maltophilia* | 11 (11.2%) | 7 (9.7%) | 0 | 1 (3.0%) | 14 (7.7%) | 1 (5.3%) |
| *Pseudomonas aeruginosa* | 4 (4.1%) | 0 | 1 (6.3%) | 1 (3.0%) | 5 (2.7%) | 0 |
| Other Non-Enterobacteriacae species | 0 | 0 | 0 | 0 | 1 (0.5%) | 0 |
| Gram-positive | 27 (27.6%) | 52 (72.2%) | 14 (87.5%) | 7 (21.2%) | 55 (30.2%) | 2 (10.5%) |
| *Staphylococcus aureus* | 7 (7.1%) | 5 (6.9%) | 7 (43.8%) | 2 (6.1%) | 11 (6.0%) | 0 |
| *Streptococcus suis* | 7 (7.1%) | 40 (55.6%) | 0 | 1 (3.0%) | 30 (16.5%) | 1 (5.3%) |
| *Streptococcus pneumoniae* | 2 (2.0%) | 1 (1.4%) | 0 | 0 | 0 | 0 |
| Beta hemolytic *Streptococcus* | 4 (4.1%) | 1 (1.4%) | 0 | 0 | 1 (0.5%) | 0 |
| Viridans group *Streptococcus* | 3 (3.1%) | 1 (1.4%) | 4 (25.0%) | 3 (9.1%) | 4 (2.2%) | 0 |
| *Enterococcus* species | 1 (1.0%) | 2 (2.8%) | 3 (18.8%) | 1 (3.0%) | 4 (2.2%) | 0 |
| Other gram-positive species | 3 (3.1%) | 2 (2.8%) | 0 | 0 | 5 (2.7%) | 1 (5.3%) |
